# A preliminary report on oral fat tolerance test in rhesus monkeys

**DOI:** 10.1186/1476-511X-13-11

**Published:** 2014-01-10

**Authors:** Di Wu, Qingsu Liu, Shiyuan Wei, Yu Alex Zhang, Feng Yue

**Affiliations:** 1 Cell Therapy Center, Beijing Institute of Geriatrics, Xuanwu Hospital, Capital Medical University, Beijing 100053, China; 2 Wincon Laboratory, Wincon TheraCells Biotechnologies Co., Ltd, Nanning, Guangxi Province 530003, China; 3 College of Light Industry and Food Engineering, Guangxi University, Nanning 530004, China; 4 Department of Neurobiology, Beijing Institute of Geriatrics, Xuanwu Hospital, Capital Medical University, Beijing 100053, China

**Keywords:** Triglyceride, Lipid metabolism, Oral fat tolerance test, Rhesus monkey

## Abstract

**Background:**

Oral fat tolerance test (OFTT) has been widely used to assess the postprandial lipemia in human beings, but there is few studies concerning OFTT in nonhuman primates. This study is designed to explore the feasibility of OFTT in rhesus monkeys.

**Methods:**

In a cross-over study, a total of 8 adult female rhesus monkeys were fed with normal monkey diet (NND), high sugar high fat diet (HHD), and extremely high fat diet (EHD), respectively. Each monkey consumed NND, HHD and EHD respectively, each weighing 60 g. Serial blood samples were collected at 1, 2, 3, 4, 5, and 6 h after ingesting each kind of food. Triglyceride, cholesterol, glucose, and insulin at each time point were measured. The area under the curve of triglyceride (TG-AUC) and triglyceride peak response (TG-PR) were also calculated.

**Results:**

All monkeys ingested 3 kinds of foods within 15 minutes. TG-AUC and TG-PR of HHD group were higher than those of the other two groups. Postprandial triglyceride levels at 2, 3, 4, and 5 hours in HHD group during OFTT were also higher than those in NND and EHD group.

**Conclusions:**

HHD diet can be used in OFTT for nonhuman primates.

## 

The diagnosis of hyperlipidemia and metabolic syndrome is generally based on fasting lipids in clinical practice. However, accumulated evidence suggests that the postprandial lipemia play an important role in the process of cardiovascular disease and type 2 diabetes [1]. We spend most part of our lives in the postprandial state, so postprandial lipemia are worth giving additional attention. In a prospective cohort study with a mean follow-up of 26 years, elevated non-fasting triglyceride (TG) levels were found to be associated with increased risk of myocardial infarction, ischemic heart disease, and death in men and women [2]. Oral fat tolerance test (OFTT) is widely used to assess the postprandial lipemia in human beings, in which an oral fat load is administered to fasting people. The exaggeration of TG was observed in patients with metabolic syndrome (MetS) when compared with normal people after consuming the same fat load [3]. It was also found that blood pressure, inflammation, oxidation, endothelia function were all affected in human beings following fat loads [4]. However, limited data is available for OFTT in nonhuman primates.

Spontaneous excessive weight gain has been observed in the middle-aged rhesus and cynomolgus monkeys in the captivity or free-ranging facilities, even with calorie restriction measures [5]. In addition, obesity could also be induced in cynomolgus and rhesus monkeys through a long-term high fat and high fructose diet [5,6]. Abnormalities in lipid metabolism have been found in overweight or obese cynomolgus and rhesus monkeys in the fasting state [7,8]. Higher values of fasting Total cholesterol (TC) and low-density lipoprotein (LDL) have also been discovered in cynomolgus monkeys when feeding a high-sugar high-fat (HSHF) diet for 33 weeks [9]. However, most studies using nonhuman primates generally paid less or even no attention to the postprandial lipemia and associated changes in the animals. In humans, obesity has become an increasingly important issue, and even obesity is considered as a disease according to American Medical Association. Nonhuman primates are excellent animal models for obesity study due to their close genetic relationship with humans, similar physiological changes and problems related to obesity. So it is necessary to explore the feasibility the OFTT in nonhuman primate.

## Materials and methods

### Animals

Eight mature female rhesus monkeys were included into this study, aging 6–7 years old, weighing 4.9-7.6 kg. They were obtained from the primate research center of Wincon TheraCells Co. (An AAALAC accredited facility in Nanning, China). These monkeys were free of TB, Shigella, Salmonella, Helminths, Ectoparasites, Entamaeba histolytica and B virus, kept for other research projects and were used in this study prior to the start of any other designated experiments. Animal use and care were conducted at the primate research center of Wincon TheraCells Co, according to the protocol approved by the Institutional Animal Care and Use Committee (IACUC). All animals were housed in controlled conditions of temperature (21-26°C), humidity (40%-70%) and lighting (7:30–20:00). The animals were fed with commercially prepared monkey food, plus fruits twice daily.

All adult females were considered to be non-pregnant via daily menses observation by feeders in the morning prior to blood sampling. Daily fruits and freely-available purified water supply were provided for these rhesus monkeys.

### Diet

Three kinds of foods were prepared for this study, including normal monkey diet (NND), high sugar high fat diet (HHD), and extremely high fat diet (EHD). NND was prepared using 98.5% commercially prepared monkey food (Foshan T&F Pet Food Co.LTD, Guangdong, China) and 1.5% water. HHD was prepared with 73% commercially prepared monkey food (Foshan T&F Pet Food Co.LTD, Guangdong, China), 7% lard, 8% vegetable oil, 10.5% sucrose, and 1.5% water according to a previous report. EHD was prepared with 73% commercially prepared monkey food (Foshan T&F Pet Food Co.LTD, Guangdong, China), 13% lard, 10% vegetable oil, 2.5% sucrose, and 1.5% water according to previous reports [6,10]. Three kinds of foods were all flavored with artificial fruit favors (TianNing flavor &fragrance.Co.LTD, Jiangsu, China) and baked to palatable pellets. Each pellet weighs 10 g.

Three kinds of foods were produced at the College of Light Industry and Food Engineering, Guangxi University. Production procedures are based on National hygienical standard for feeds (GB13078-2001).

### Oral fat tolerance test

The OFTT procedures in this study were referring to those in human beings. All monkeys underwent an OFTT after a 16 h fast in the morning. Firstly, they were lightly anesthetized with ketamine (5 mg/kg, im). Fasting blood was collected into plain tubes on ice within 30 minutes prior to centrifugation. After a recovery of approximately 20 minutes, they were fed with NND, HHD, or EHD. Each animal was fed with 6 pieces of pellets (60 g totally), and the food should be ingested within 15 minutes. When animals did not consume the required food in quantity, or the meal time was longer than 15 minutes, the experiment was suspended and it was carried out again in the second day. The repeated blood samples were drawn at 1, 2, 3, 4, 5, and 6 hours after each kind of meal. They were also lightly anesthetized with ketamine (5 mg/kg, im) before each time point. The experiment with NND was finished within 2 weeks, HHD and EHD followed.

Biochemistry measurements were performed on serum samples, using a Mindray BS 420 autoanalyzer (Mindray DS, Shenzhen, China), including TG (mmol/L), TC (mmol/L), glucose (GLU, mmol/L). Insulin (INS, mIU/ml) was measured on serum sample using a Chemclin®600 analyzer with enhanced chemiluminescence immunoassay (Chemclin Biotech co., Ltd., Beijing, china).

Areas under the curve (AUC) for serial measurements of TG, TC, GLU, and IN levels at baseline and after each meal were calculated using the trapezoid rule. The increase of TG levels from the 0-hour sample to the mean of the two highest triglyceride values found in postprandial state was calculated as triglyceride peak response (TG-PR) based on the formula: TG-PR = ( n_max_ + n_2nd_ )/2 – n_0_, in which n_max_ is the highest level, n_2nd_ the second highest level, and n_0_ the 0-hour triglyceride value [11].

#### Statistical analysis

All data are expressed as mean ± SD. Statistical differences among three groups were analyzed by one-way ANOVA using the SNK test for post-hoc comparisons after assessment of normality of data distribution. Student’s t-test was applied to compare fasting level with postprandial levels. Statistical difference was defined as P<0.05.

## Result

All monkeys ingested 3 kinds of foods within 15 minutes, in which only 2 animals were reluctant to eat EHD. When the flavor of EHD was changed, experiments were successful on the second day.

### The curves of TG

**Figure 1:**
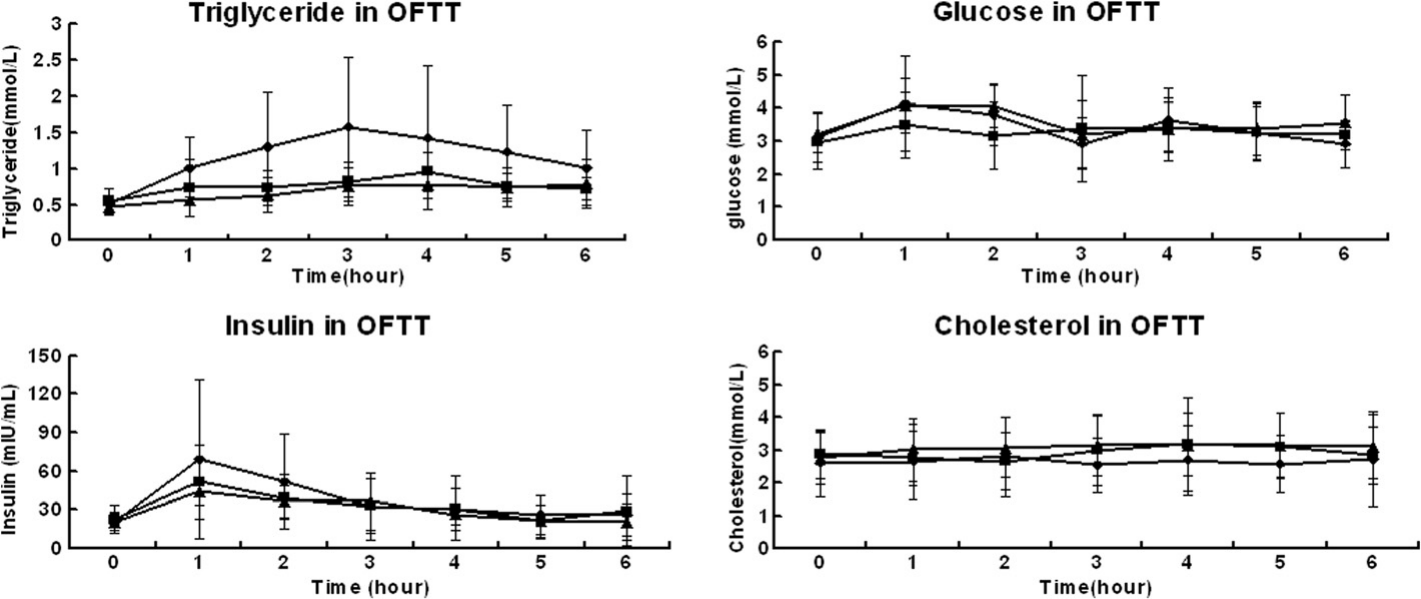
The curves of triglyceride, glucose, insulin and cholesterol during oral fat tolerance test, in which black square stands for normal monkey diet (NND), black rhombus stands for high sugar high fat diet (HHD), black triangle stands for extremely high fat diet (EHD).

The highest postprandial TG level occurred at the 3 or 4 hours after the diet when compared with the fasting level in each food group as shown in Table 1. TG-AUC and TG-PR of HHD group were higher than those of the other two groups according to Table 1. As illustrated in Figure 1, postprandial TG levels at the 2, 3, 4, and 5 hours following the diet in HHD group were higher than those of NND and EHD group.

**Table 1:** Postprandial triglyceride responses in OFTT

**Group**	**Percentage of baseline (0 h) variation**						**TG-AUC**	**TG-PR**
	**1 h**	**2 h**	**3 h**	**4 h**	**5 h**	**6 h**	**mmol/L × 6 h**	
NND	135	132	149 ^a^	174 ^a^	136 ^b^	130	1.43 ± 0.66 ^c^	0.44 ± 0.18 ^c^
HHD	200 ^a^	254 ^a^	308 ^a^	278 ^a^	241 ^a^	198 ^a^	3.99 ± 3.29	1.21 ± 0.85
EHD	121	136	162 ^a^	166 ^a^	147 ^a^	168 ^a^	1.26 ± 0.63 ^d^	0.42 ± 0.21 ^d^

TG-AUC stands for area under the curve of triglyceride during OFTT, and TG- PR for peak response of triglyceride during OFTT. Data as mean + SEM; a and b: differences from 0 h, ^a^P<0.05, ^b^P<0.01. c and d: differences from HHD group, ^c^P<0.05, ^d^P<0.01.

### The curves of TC and GLU

As illustrated in Figure 1, the highest postprandial glucose level occurred at 1 hour following the diet in three groups. When GLU-AUC was compared among three groups, there was no significant difference according to Table 2. Postprandial GLU levels at the 1 and 2 hours following the diet in HHD and EHD groups were higher than those of NND group at the same time points.

**Table 2:** Postprandial cholesterol, glucose and insulin responses during OFTT

**Group**	**TC-AUC**	**GLU-AUC**	**INS-AUC**
	**mmol/L × 6 h**	**mmol/L × 6 h**	**mIU/mL × 6 h**
NND	0.57 ± 2.40	0.90 ± 5.00	59.4 ± 57.0
HHD	0.72 ± 3.19	2.76 ± 4.53	90.6 ± 97.3
EHD	1.76 ± 2.22	2.23 ± 3.07	43.0 ± 44.3

TC-AUC stands for area under the curve of cholesterol, GLU-AUC for area under the curve of glucose, and INS-AUC for area under the curve of insulin.

The TC curve was near to a horizontal line without obvious higher level point. There were also no significant differences in TC-AUC among three groups.

It was also observed during experiments that some GLU and TC levels following three diets were lower than those of fasting levels. So, some GLU and TC curves formed a saddle type.

### The curves of insulin

As shown in Figure 1, the highest postprandial insulin level occurred at 1 hour following the diet in three groups. When INS-AUC was compared among three groups, there were no significant differences despite a relatively higher INS-AUC in HHD group according to Table 2. There were also no significant differences when insulin levels at each time point were compared among three groups.

## Discussion

OFTT has been widely used to evaluate the postprandial lipemia in both human and rodent animals. In a previous report, the responses in mice following OFTT were closer to those in healthy men [12]. But, there are different features of lipid metabolism between human and rodent, such as LDL as transporter of TC in human, but HDL as the transporter in mice [13]. Lipid metabolism between nonhuman primate and human are generally the same, with monkeys in a lower level, such as TG, TC, HDL, and LDL, with human in a lower level, such as insulin [14]. Therefore, OFTT in nonhuman primates will provide more information to human research. However, few papers are designed to assess the postprandial lipemia in nonhuman primates. The monkeys’ compliance to the test and diet choice may be the main concerns for OFTT in nonhuman primates. This study adopted a cross-over design to find the postprandial responses following three kinds of diet challenges. This study may be the first research to explore the feasibility of OFTT in nonhuman primates.

There was no consensus on guidelines for OFTT procedures in humans, so various studies adopted different fat diets both in the composition and in the energy intake. Milkshake consisting of whipping cream, vanilla ice cream and syrup was adopted as the fat meal for humans in a study [1]. In another study, milk cream was used as fat food for rodents, and milkshake prepared with lactose-free powdered milk was used as the fat food for humans [12]. So it is difficult to consult a standardized oral fat diet based on existing studies. Generally, nonhuman primates consume a low fat diet in the facilities compared with human diet [5]. High fat and high glucose diet has been applied to induce obesity in nonhuman primates by adding lard, vegetable oil and fructose [9]. In anther study, high fat and high fructose diet for cynomolgus monkeys contained 15% lard and 31% fructose. However, the fat percentage of high fat and high fructose diet for nonhuman primates (18.5% fat) is lower than that of high fat diet for humans (50% fat) [9,15]. So we chose three kinds of foods in this study, NND made by commonly used monkey chow as control, HHD based on commonly used high fat and high glucose diet for nonhuman primates, and EHD by combination of monkey chow and a higher content of fat.

In addition to diet composition, food shape and quantity are anther concerns for OFTT in nonhuman primate. Monkey chow was generally little pellets containing balanced nutrients in the facility. But, many pellets were lost through their fingers or due to their naught nature when monkeys grabbed the food. So it was difficult to quantify the intake for the monkey in an accurate way with the existing monkey chow. Based on our observation and pilot experiment, rhesus monkeys, aging 6–7 years old, can easily take in a 10 g pellet. Therefore, monkey chow weighing 10 g can help us to complete OFTT in a quantitative way. OFTT shares many similarities with oral glucose tolerance test (OGTT), such as a challenge load, corresponding lipid or glucose rise, and physiological and biochemistry responses following the challenge [16]. A standard dose of glucose is widely adopted during oral glucose tolerance test in humans, so we also select a standard dose of diet in this study. All monkeys could take in 60 g each kind of diet within 15 minutes. So it is feasible to perform OFTT in nonhuman primates according to this study. But, the physiological response to a diet may be more complex than that to a simple glucose bolus.

Lipid changes following diet challenges in nonhuman primates were similar to those of humans. TG levels were increased in all animals following each kind of food, and TC levels generally remained unchanged, which were also observed in human studies [17]. Oral fat load could resulted in a dose-dependent increase in TG levels after low and high fat load in obese healthy subject [4], which was also observed in this study when comparing TG levels between NND and HHD group. Based on the above results, it was assumed before experiments that postprandial TG level and TG-AUC of EHD group would be higher than those of the other two groups because of higher content of fat in the diet. But based on our observation, postprandial TG levels and TG-AUC of EHD group were lower than those of HHD group, showing no significant differences from NND group. Various factors, such as the type, nature and amount of fat in the diet, plasma lipidome response following a lipid challenge, metabolic changes, have been found to influence postprandial metabolism following a dietary perturbation [18,19]. But, it was highly possible that rhesus monkeys were accustomed to a low-fat monkey chow, and when diet was suddenly changed to EHD, rhesus monkeys could not make a timely adjustment. So it may not be a preferable choice of selecting EHD for OFTT in nonhuman primate.

TC and GLU levels following consuming three diets were increased or decreased when compared with the fasting levels, so that means of TC-AUC and GLU-AUC were lower than standard deviations. TC levels were found to generally remain unchanged, and GLU with a peak level at 1 or 2 hour after the diet. The above findings were similar to changes of TC and GLU in human study following diet challenge [15,20]. Higher levels of insulin secretion during OFTT in HHD group may be due to relatively higher glucose and triglyceride levels in this group. Higher insulin release was found in healthy and obese humans after oral fat load and glucose challenge when compared with only glucose challenge [21]. Insulin may be the primary responding factor in both glucose and diet challenge. But compared with the simple glucose challenge in OGTT, the composition of a diet in OFTT was more complex, therefore physiological and biochemical response to a diet may be more complicated, including energy storage, metabolic switches in liver, muscle and adipose tissue, inflammation and oxidation, endothelial function alterations [16].

That OFTT only last 6 hours without obvious TG decrease trend is one of the limitations in this study. Another limitation is that only adult female rhesus monkeys are recruited into this study without inclusion adolescent, aged and male rhesus monkeys.

## Conclusion

It is feasible to perform OFTT in nonhuman primates with commonly used HHD diet. When the fat content is too high, the postprandial TG levels will not indicate a dose-dependent increase.

## Competing interests

The authors declare that they have no competing interests.

## Authors’ contribution

Conduct of the study: DW, QL, YW. Design and manuscript writing: DW, YZ, FY. Data collection and analysis: DW and FY. All authors have read and approved the final manuscript.
